# Opinion on Updating the Taxonomic Names of Aspergillus terreus Strains IFO 6365 and TN-484

**DOI:** 10.1128/MRA.00472-21

**Published:** 2021-12-02

**Authors:** Shin Kanamasa

**Affiliations:** a College of Bioscience and Biotechnology, Chubu University, Kasugai, Aichi, Japan; Vanderbilt University

## REPLY

In their letter to the editors, Dr. Houbraken et al. pointed out that the correct identification of a fungal strain is important and reports with inaccurate strain identification confuse researchers (1). They also proposed a procedure to prevent this issue of inaccurate strain identification. I totally understand the reason for raising this issue and the need for the proposed procedure to ensure accuracy. Therefore, I wish to respond to the points highlighted in the letter. It was incorrect to claim that strain IFO 6365 (2) and its mutant strain, TN-484 (3), were Aspergillus terreus; these were consistently and correctly identified as Aspergillus pseudoterreus. Although I agree with the importance of listing fungal strains accurately, there is also a need to stress the importance of consistency among all our previous reports to ensure the reader’s understanding.

First and foremost, I would like to clarify the identity of strain IFO 6365 (=NBRC 6365). In actuality, this strain was not isolated and identified by our research team but was instead obtained from an established microbial preservation institution in Japan. This particular strain was initially registered in 1958 with the Institute for Fermentation, Osaka (IFO), Japan, a former institution focused on research related to microbial conservation; this strain was subsequently transferred to the Biological Resource Center, National Institute of Technology and Evaluation (NBRC), and has since been distributed as strain NBRC 6365 (4). Therefore, it is not possible to change the strain name at our own discretion; we shall discuss the notation of this fungal strain with the relevant taxonomic experts at NBRC. Meanwhile, strain TN-484 was obtained by introducing a mutation into strain IFO 6365; hence, the taxonomic classification of TN-484 should be identical to that of strain IFO 6365.

Second, I would like to stress the importance of consistency in fungal strain notation in all our reports. We have published numerous articles on strains IFO 6365 and TN-484 since 1993 (5). Additionally, *A. pseudoterreus* strain NRRL 4017, as mentioned in the Comment Letter (1), was only listed in 2011 (6). The Agricultural Research Service (NRRL) Culture Collection catalog (version as of summer 2021) specifies that a synonym of strain NRRL 4017 is Aspergillus terreus Thom. Most researchers understand that the strain IFO 6365 that is described in past and present reports is the same strain, and to ensure clarity and consistency, the synonym shall be included in our future reports.
